# Provision and utilization of maternal health services during the COVID-19 pandemic in 16 hospitals in sub-Saharan Africa

**DOI:** 10.3389/fgwh.2023.1192473

**Published:** 2023-10-31

**Authors:** Aline Semaan, Kristi Sidney Annerstedt, Lenka Beňová, Jean-Paul Dossou, Christelle Boyi Hounsou, Gottfried Agballa, Gertrude Namazzi, Bianca Kandeya, Samuel Meja, Dickson Ally Mkoka, Anteneh Asefa, Soha El-halabi, Claudia Hanson

**Affiliations:** ^1^ Department of Public Health, Institute of Tropical Medicine, Antwerp, Belgium; ^2^ Department of Global Public Health, Karolinska Institutet, Stockholm, Sweden; ^3^ Department of Health Policy and Systems, Centre de Recherche en Reproduction Humaine et en Démographie, Cotonou, Benin; ^4^ Centre of Excellence for Maternal Newborn and Child Health, Department of Health Policy Planning and Management, School of Public Health, Makerere University, Kampala, Uganda; ^5^ College of Medicine, the Centre for Reproductive Health, University of Malawi, Blantyre, Malawi; ^6^ Department of Clinical Nursing, Muhimbili University of Health and Allied Sciences, Dar es Salaam, Tanzania; ^7^ Department of Disease Control, London School of Hygiene and Tropical Medicine, London, United Kingdom

**Keywords:** referral hospital, maternal health, routine data, health facility survey, COVID-19 pandemic, sub-Saharan Africa

## Abstract

**Objective:**

Maintaining provision and utilization of maternal healthcare services is susceptible to external influences. This study describes how maternity care was provided during the COVID-19 pandemic and assesses patterns of service utilization and perinatal health outcomes in 16 referral hospitals (four each) in Benin, Malawi, Tanzania and Uganda.

**Methods:**

We used an embedded case-study design and two data sources. Responses to open-ended questions in a health-facility assessment survey were analyzed with content analysis. We described categories of adaptations and care provision modalities during the pandemic at the hospital and maternity ward levels. Aggregate monthly service statistics on antenatal care, delivery, caesarean section, maternal deaths, and stillbirths covering 24 months (2019 and 2020; pre-COVID-19 and COVID-19) were examined.

**Results:**

Declines in the number of antenatal care consultations were documented in Tanzania, Malawi, and Uganda in 2020 compared to 2019. Deliveries declined in 2020 compared to 2019 in Tanzania and Uganda. Caesarean section rates decreased in Benin and increased in Tanzania in 2020 compared to 2019. Increases in maternal mortality ratio and stillbirth rate were noted in some months of 2020 in Benin and Uganda, with variability noted between hospitals. At the hospital level, teams were assigned to respond to the COVID-19 pandemic, routine meetings were cancelled, and maternal death reviews and quality improvement initiatives were interrupted. In maternity wards, staff shortages were reported during lockdowns in Uganda. Clinical guidelines and protocols were not updated formally; the number of allowed companions and visitors was reduced.

**Conclusion:**

Varying approaches within and between countries demonstrate the importance of a contextualized response to the COVID-19 pandemic. Maternal care utilization and the ability to provide quality care fluctuated with lockdowns and travel bans. Women's and maternal health workers' needs should be prioritized to avoid interruptions in the continuum of care and prevent the deterioration of perinatal health outcomes.

## 1. Introduction

The COVID-19 pandemic, declared by the World Health Organization (WHO) in March 2020, continues to challenge health systems globally. Besides responding to and managing the disease itself, health systems have struggled to maintain the provision of essential services during this period (1). In maternal and newborn healthcare, the pandemic has disrupted the availability, utilization, and quality of care provided to women and newborns (2–5). According to WHO's national pulse survey, antenatal and postnatal care (PNC) were disrupted in over a third of 121 countries, and a quarter of countries reported disruptions in facility-based births (6). In eight sub-Saharan African countries, significant reductions in the number of antenatal care (ANC) visits, facility-based childbirths, and PNC visits during the pandemic were reported (7). A survey of 500 pregnant women during the first lockdown in France (March–May 2020) revealed that one fifth had delayed or cancelled at least one ANC consultation (8). The COVID-19 pandemic also affected maternal and newborn health outcomes. There is evidence that infection with SARS-CoV-2 is associated with higher risks of stillbirths and preterm births (9). Additionally, some trends suggest increases in stillbirths and maternal deaths in low- and middle-income countries (LMIC) as a result of delayed care seeking (10).

From the supply side, maintaining care provision in healthcare facilities was challenging as health system governance and financing prioritized the response to the pandemic. Additionally, facilities were affected by national restriction measures such as lockdowns, bans on public transportation, and the emergency response to the pandemic. Some hospitals and healthcare centers were closed or converted into COVID-19 treatment centers. Pre-existing shortages in the health workforce were exacerbated by high rates of illness among providers, reassignment of healthcare workers to pandemic-related response, and implemented mitigation measures, which prevented healthcare providers from reaching the workplace (2, 11, 12). The lack of accessibility to clinical guidelines and training in a formal manner, particularly regarding care provision to women diagnosed with COVID-19, worsened fear and anxiety among midwives, nurses and doctors globally (2, 3, 11, 13–16).

Healthcare facilities globally, particularly hospitals, adapted their processes and guidelines in response to the dynamic situation resulting from the COVID-19 pandemic. Many of these adaptations aimed to ensure the safety of staff and patients, and were established, communicated and implemented relatively early in the response to the pandemic. In facilities providing maternity care, these included the allocation of triage areas for screening and testing women and establishing isolation wards to host women suspected/confirmed with COVID-19 (2, 17). A shift to telehealth was utilized to continue providing ANC (18). However, some of these adaptations were not evidence-based considering the lack of knowledge during the early phase of the pandemic. In some settings, women were required to leave healthcare facilities early after childbirth, some women were not allowed companions during childbirth, and new visiting rules restricted family and friends from accompanying mother and baby. Women with COVID-19 were not allowed to breastfeed or to be in contact with their baby (2, 19–21). In June 2020, the WHO issued guidance to ensure the continuity of provision of essential care services, including antenatal, intrapartum and PNC for the mother and newborn (22). The guide highlighted the necessity of maintaining breastfeeding and non-separation for all mothers and newborns (23).

A recently published scoping review concluded that preparedness and response to the COVID-19 pandemic in African countries was sub-optimal (24). A comprehensive assessment of trends in maternal care utilization, provision, and health outcomes during the COVID-19 pandemic is lacking, particularly in referral hospitals in LMICs. The triangulation of such quantitative trends with information on how healthcare facilities responded to the pandemic and adapted care provision processes is not available. This study aims to describe how maternity care was provided and organized, and to assess patterns of service utilization and perinatal health outcomes, before and during COVID-19 in four referral hospitals each in Benin, Malawi, Tanzania and Uganda.

## 2. Methods

### 2.1. Context

This study is part of the Action Leveraging Evidence to Reduce perinatal mortality and morbidity in sub-Saharan Africa trial (ALERT) (25). The project aims to reduce perinatal mortality and morbidity by strengthening the health system to provide safe and respectful intrapartum care. It is conducted in four hospitals each in four countries (Benin, Malawi, Tanzania, and Uganda). From each country, there are three public hospitals and one private/faith-based. All 16 hospitals provide outpatient ANC and care for vaginal births and caesarean sections. In 2019, the number of deliveries ranged from 1,265 in UG2 to 7,791 in UG3. The 2019 perinatal mortality rate ranged from 17 perinatal deaths per 1,000 births in MW2 to 115 perinatal deaths per 1,000 births in UG2 (Supplementary File S1).

Each of the four countries experienced the COVID-19 pandemic differently. Supplementary File S2 summarizes response measures and their respective duration during 2020, as well as the daily number of confirmed COVID-19 cases during the same time period. Data on response measures were extracted from Oxford COVID-19 Government Response Tracker (26) and validated by the ALERT country teams. We selected response measures which potentially influence healthcare-seeking behaviors and the ability to provide services, such as movement restrictions, stay-at-home orders, public transportation bans, and school and workplace closures. Data on the number of COVID-19 cases were extracted from the WHO COVID-19 dashboard (27). In Benin, strict measures of movement restrictions and school closure were implemented shortly between April–May 2020, without staying at home requirements. Restricting the number of passengers allowed in public transport was applied between April–June 2020. In Malawi, schools closed between March–September 2020, without any strict measures regarding movement restrictions, staying-at-home requirements and public transportation bans. In Tanzania, schools closed between March–June 2020, and international travel was restricted. Country authorities stopped reporting COVID-19 statistics to WHO since 4 July 2020. Uganda had the longest period of response measures, with school closures and movement restrictions implemented between March–October 2020. Public transportation closed between March-September 2020, and a stay-at-home requirement was issued from April 2020 until the end of the study period.

### 2.2. Study design

This is an embedded case study, where the units of analysis are hospitals during the COVID-19 pandemic, and the sub-units are the maternity wards in each hospital (28, 29). We use data from two sources: data of routinely collected maternal and perinatal health indicators; and responses to open-ended questions in an in-depth health facility assessment (HFA). Collection, analysis and reporting of both data types were conducted independently. The results were integrated during the interpretation phase. We use the framework for organizational case studies as a reporting checklist (30).

### 2.3. Data collection and measures

This study uses data from a maternity-oriented HFA intended to collect baseline data for the ALERT project. The original questionnaire included a mix of closed- and open-ended questions on hospital governance; financing; infrastructure and supplies; human resources; medicine availability; laboratory support; and guidelines, standards and practices for care provision. Data collection was conducted between December 2020–April 2021 by senior researchers and trained data collectors (at least two per hospital) who were familiar with the participating hospitals. The HFA took between two-three days per hospital to collect and relied on interviews, notes, observations, GPS coordinate logging, and document reviews. An average of three, but up to six respondents were interviewed per hospital. All data were entered into REDCap (31). Additional details about HFA data collection are available elsewhere (32).

The questionnaire included collecting aggregate monthly service statistics covering 24 months between January 2019–December 2020. These data were retrieved from each hospital's Health Management Information System (HMIS) and entered onto REDCap. A quality check allowed the identification of outliers and missing values, which were shared with ALERT country teams who thoroughly verified the data in the HMIS. A review of facility registers was not conducted, partly attributed to the complexity of in-hospital documentation systems (32). Five indicators were selected for this analysis based on data availability, completion, and accuracy across the 16 hospitals: (1) number of outpatient ANC consultations; (2) number of deliveries (women who gave birth); (3) number of caesarean sections and percentage of caesarean section out of deliveries; (4) number of maternal deaths and the ratio of maternal deaths per 100,000 deliveries; and (5) number of stillbirths and rate of stillbirths per 1,000 deliveries. For the last two indicators, the number of deliveries was used as a denominator to calculate the ratio and rate due to the unavailability of highly accurate disaggregated data on the number of births (stillbirths and livebirths) in all 16 hospitals.

The HFA data collection period (December 2020–April 2021) coincided with the COVID-19 pandemic, which presented an opportunity to incorporate brief questions in the questionnaire on the pandemic's effect on participating hospitals in general and on maternity wards in particular. We added open-ended questions on perceived changes in governance/financing (including creation of committees and budgetary changes), infrastructure/supplies (triage zones and/or isolation areas/rooms), staffing (number, cadres, trainings), changes to guidelines and protocols (breastfeeding, separation, visitors and companions, discharge), and changes to the implementation of quality improvement (QI) initiatives and maternal and perinatal death reviews. Additionally, we asked about specific changes regarding the number of women with suspected/confirmed SARS-CoV-2 infection and how care was provided to them (33).

### 2.4. Data analysis

The five indicators from service statistics were combined from each of the four hospitals per country. We conducted descriptive analysis and presented the data in bar and line charts, separately for 2019 and 2020, and compared the values between the months of both years. Indicators on maternal deaths and stillbirths heavily fluctuated month-to-month due to the low frequency of the outcome, we therefore present them summarized by quarter (three months). Some values were completely missing and inaccessible from the HMIS, including data on ANC from two hospitals in Tanzania and one in Uganda. These hospitals were excluded from the analysis of this specific indicator.

Data on perceived changes made in hospitals and maternity wards during the pandemic were obtained in textual form, to which we applied content analysis (34). The researchers read and re-read the data by hospital (or case). An analysis framework was developed using categories derived from the HFA sections (deductive approach). Relevant information from the open-text was identified, extracted, and classified into corresponding categories of the framework. As data extraction progressed, the content of the framework evolved to include new categories that were identified in the data (iterative inductive approach). Disagreements and uncertainties were discussed between researchers including country teams, and resolved by consensus. Extracted information was compared between the 16 hospitals and patterns were identified. Findings were summarized in a narrative format, and presented by category at the hospital level and maternity ward level.

### 2.5. Ethics

Ethical approval was granted by review boards of the authors’ institutes. No individual data were collected for this study, therefore individual informed consent was not required. With data collection conducted during the COVID-19 pandemic, each country followed the risk protocol put in place by their respective ethics committees in the beginning of the pandemic. These protocols were followed in relation to all research activities until further notified by the ethics committees. Throughout the manuscript, we refer to participating hospitals using a random coding system (country and hospital number) to protect the identity of hospitals and their staff.

## 3. Results

### 3.1. Routine service statistics

In the following section, we summarize the results of the routine service statistics, aggregated by country, for each of the following indicators: ANC visits, number of deliveries, proportion of caesarean section, maternal deaths, and stillbirths.

#### 3.1.1. Antenatal care

**Figure 1:**
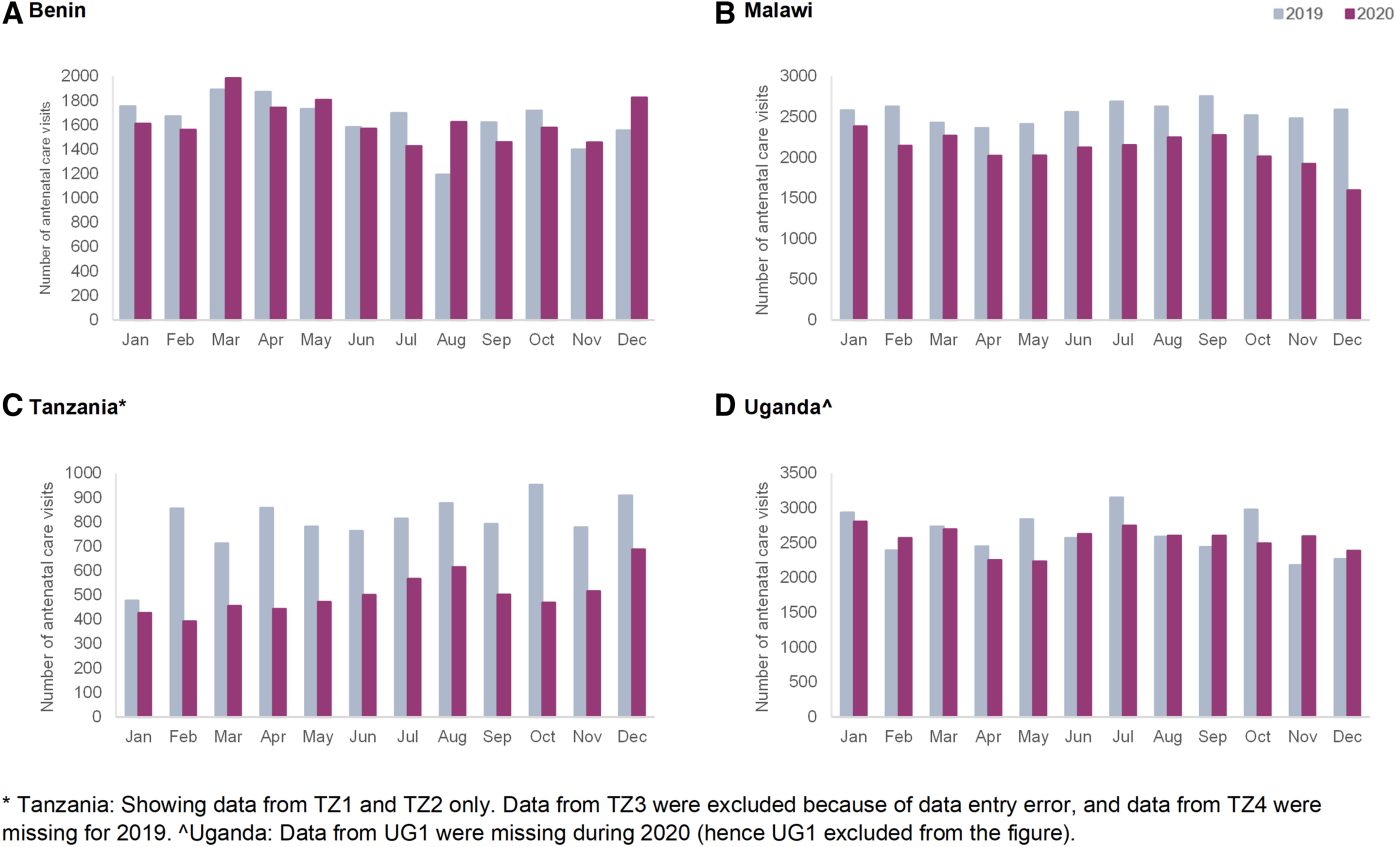
Monthly number of antenatal care consultations in the four hospitals in 2019 and 2020 in (**A**) Benin, (**B**) Malawi, (**C**) Tanzania and (**D**) Uganda.

Figure 1 displays routine data on outpatient ANC consultations, by country. In Benin, the number of consultations ranges between 1,195 in August 2019 and 1,984 in March 2020. There was no reduction in ANC visits during the pandemic compared to the previous year in Benin hospitals; on the contrary, the number of consultations in some months of 2020 exceeded that of 2019 (e.g., August 2020 in Benin). In Malawi, the minimum number of consultations was 1,597 in December 2020, and the maximum was 2,754 in September 2019. In Tanzania, the number of ANC consultations ranged between 393 in February 2020, and 953 in October 2019. In Malawi and Tanzania, the number of ANC consultations was lower in all months of 2020 compared to those of 2019. In Uganda, the number of ANC visits ranged between 2,184 and 3,155 in November and July 2019, respectively. The number of ANC visits in 2020 was similar to 2019, with the exception of a decline observed in April and May 2020 compared to the same months of 2019.

#### 3.1.2. Deliveries

**Figure 2:**
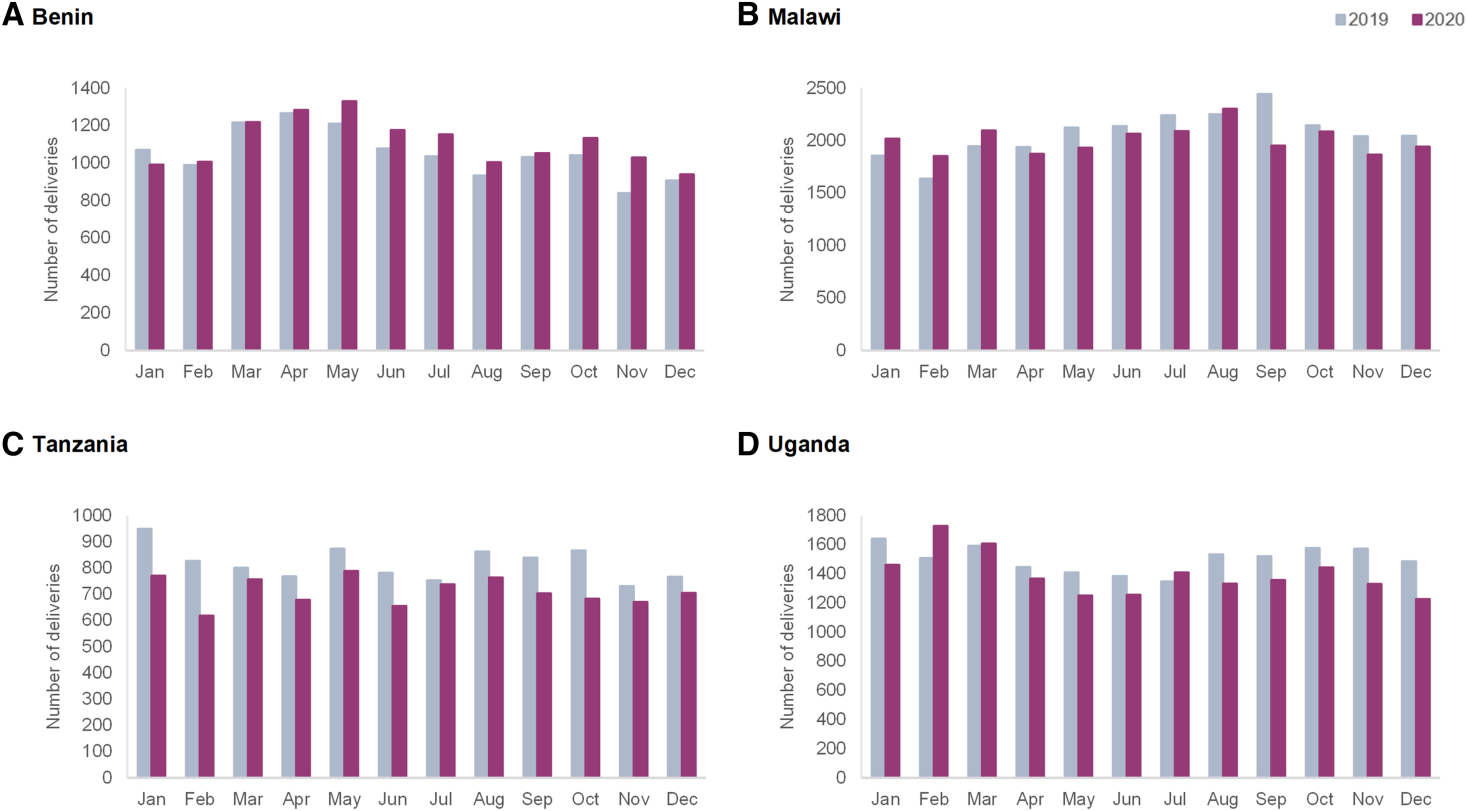
Monthly number of deliveries in the four hospitals in 2019 and 2020 in (**A**) Benin, (**B**) Malawi, (**C**) Tanzania and (**D**) Uganda.

Figure 2 presents the total number of deliveries, combined by country. In Benin, the monthly number of deliveries ranged between 840 and 1,329 over the study period, and there were no big differences between 2019 and 2020. In Malawi, the number of deliveries ranged between 1,635 and 2,442 per month. The number of deliveries remained stable in 2020 compared to 2019, with the exception of a decline in the number of deliveries in September 2020 compared to 2019. In Tanzania, the number of deliveries ranged between 618 and 949 per month, and lower values were observed in all months of 2020 compared to 2019. In Uganda, the number of deliveries ranged between 1,226 and 1,728, and a small decline in the number of deliveries was observed starting in April 2020 compared to 2019.

#### 3.1.3. Caesarean section

**Figure 3:**
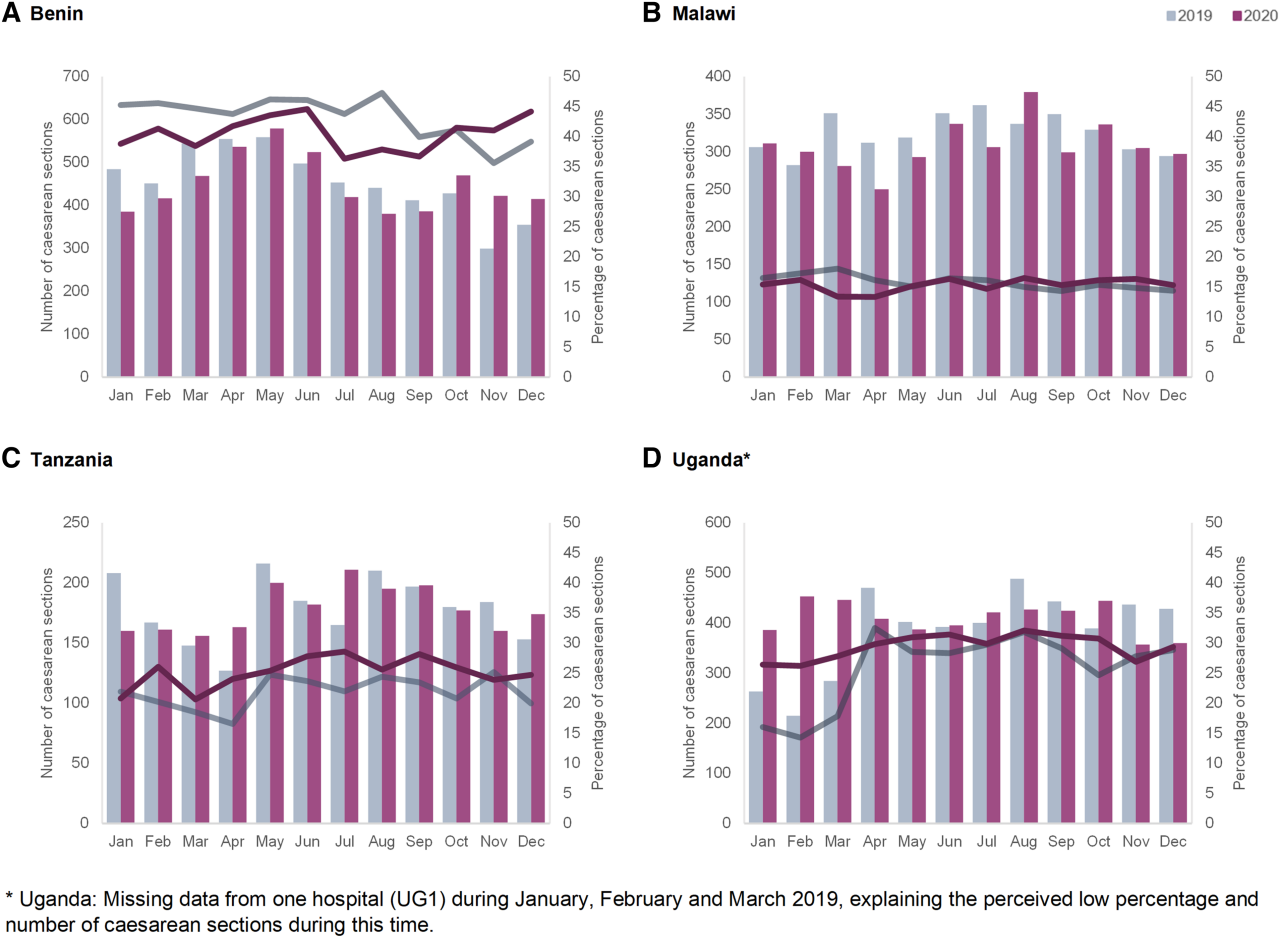
Monthly numbers (bars—left axis) and percentages (lines—right axis) of caesarean section in the four hospitals in 2019 and 2020 in (**A**) Benin, (**B**) Malawi, (**C**) Tanzania and (**D**) Uganda.

Figure 3 shows the caesarean section rates by country. Monthly rates ranged from 13% in Malawi to 47% in Benin. In Benin, the percentage of caesarean sections was lower during the year 2020 compared to 2019. In Malawi, the caesarean section rate was comparable between the COVID-19 period and the year before. In Tanzania, there was a small increase in the percentage of deliveries by caesarean sections from April to October 2020 compared to 2019. In Uganda, the monthly proportion of caesarean sections was similar in 2020 compared to 2019, with the exception of an increase observed in September 2020.

#### 3.1.4. Maternal deaths

**Figure 4:**
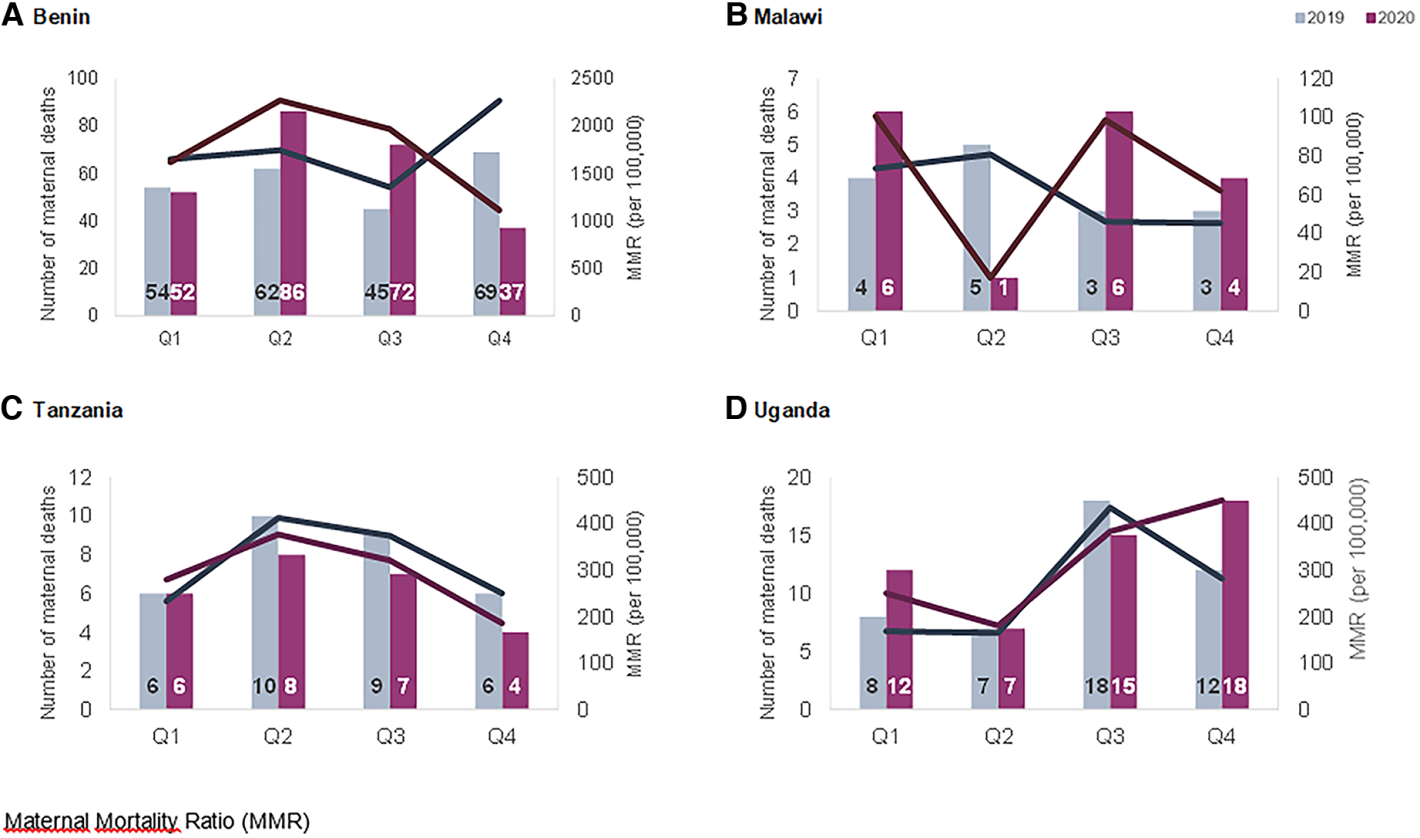
Quarterly number (bars—left axis) and in-facility maternal mortality per 100,000 deliveries (lines—right axis) in the four hospitals in 2019 and 2020 in (**A**) Benin, (**B**) Malawi, (**C**) Tanzania and (**D**) Uganda.

Figure 4 shows the quarterly numbers of maternal deaths and the in-facility maternal mortality ratio per 100,000 deliveries in 2019 and 2020. The highest ratio was recorded in Benin in Q2 of 2020 reaching 2,265.3 maternal deaths per 100,000 deliveries. The lowest rate is noted in Malawi in Q2 of 2020 with 17 maternal deaths per 100,000 deliveries (Table 1). In Benin, there was a small increase observed in Q2 and Q3 in 2020 compared to 2019, followed by a decline in Q4 2020. In Malawi and Tanzania, the ratio of maternal deaths remained stable between 2019 and 2020. In Uganda, it was constant between 2019 and 2020, with an increase observed in the last quarter of 2020 compared to the same period in 2019.

**Table 1:** In-facility maternal mortality per 100,000 deliveries, and stillbirth rates per 1,000 deliveries, by quarters in the four hospitals in Benin, Malawi, Tanzania and Uganda.

		2019				2020			
		Q1	Q2	Q3	Q4	Q1	Q2	Q3	Q4
Maternal mortality ratio	Benin	1,647.8	1,744.5	1,354.2	2,265.3	1,616.9	2,269.7	1,968.3	1,110.1
	Malawi	73.6	80.6	46.1	45.2	100.5	17.0	98.6	62.0
	Tanzania	232.8	412.7	373.9	250.4	279.6	376.8	320.8	185.5
	Uganda	168.8	165.1	434.7	281.3	250.3	180.6	382.8	450.1
Stillbirth rate	Benin	81.8	63.6	52.4	66.0	61.6	64.7	70.5	79.2
	Malawi	29.2	14.2	20.4	20.7	15.1	17.0	21.0	16.7
	Tanzania	28.7	28.5	29.1	28.4	26.1	23.6	16.5	26.9
	Uganda	^a^	47.6	46.8	49.9	45.7	44.4	56.9	54.8

^a^ Data on stillbirths in Q1 of 2019 in Uganda was removed from the analysis due to data quality issues.

#### 3.1.5. Stillbirths

**Figure 5:**
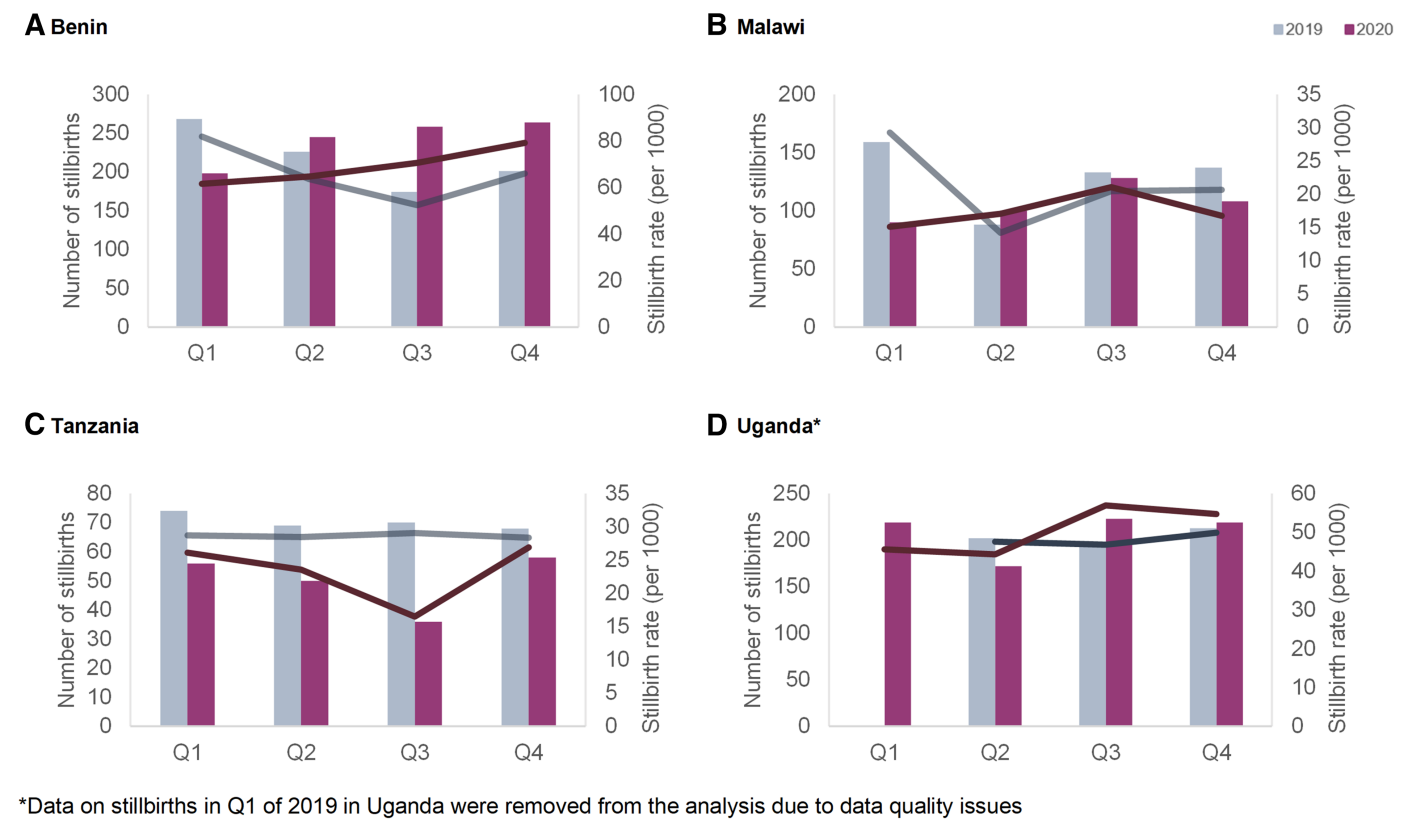
Quarterly number (bars—left axis) and rate (lines—right axis) of stillbirths per 1,000 deliveries in the four hospitals in 2019 and 2020 in (**A**) Benin, (**B**) Malawi, (**C**) Tanzania and (**D**) Uganda.

Figure 5 displays the quarterly stillbirth number and rate per 1,000 deliveries in 2019 and 2020, per country. The highest rate was recorded in Benin in Q1 of 2019 reaching 82 stillbirths per 1,000 deliveries; the lowest value was in Malawi with 14 stillbirths per 1,000 deliveries in the second quarter of 2019 (Table 1). There was an increase in the stillbirth rate in Benin and Uganda at one point in time in 2020 compared to 2019: this increase occurred in Benin during the third and fourth quarter and in Uganda during the third and fourth quarters. In Malawi, the proportion of stillbirths was lower in the first quarter of 2020 compared to 2019, and similar throughout the remaining quarters. In Tanzania, the proportion of stillbirths was lower in 2020 compared to 2019.

Hospital-level data on the selected indicators are available in Supplementary File S3 as well as a heatmap showing the percentage change in indicators between 2020 and 2019, for each hospital and country. The disaggregation shows variability in trends between hospitals in the same country during the study period. For example, while two hospitals (MW1 and MW4) show an increase in MMR in 2020, one hospital (MW3) shows a decline. In Benin, stillbirths increased in one hospital (BN2), and declined in two others (BN1 and BN4).

### 3.2. Management and organization of health services during the COVID-19 pandemic

**Figure 6:**
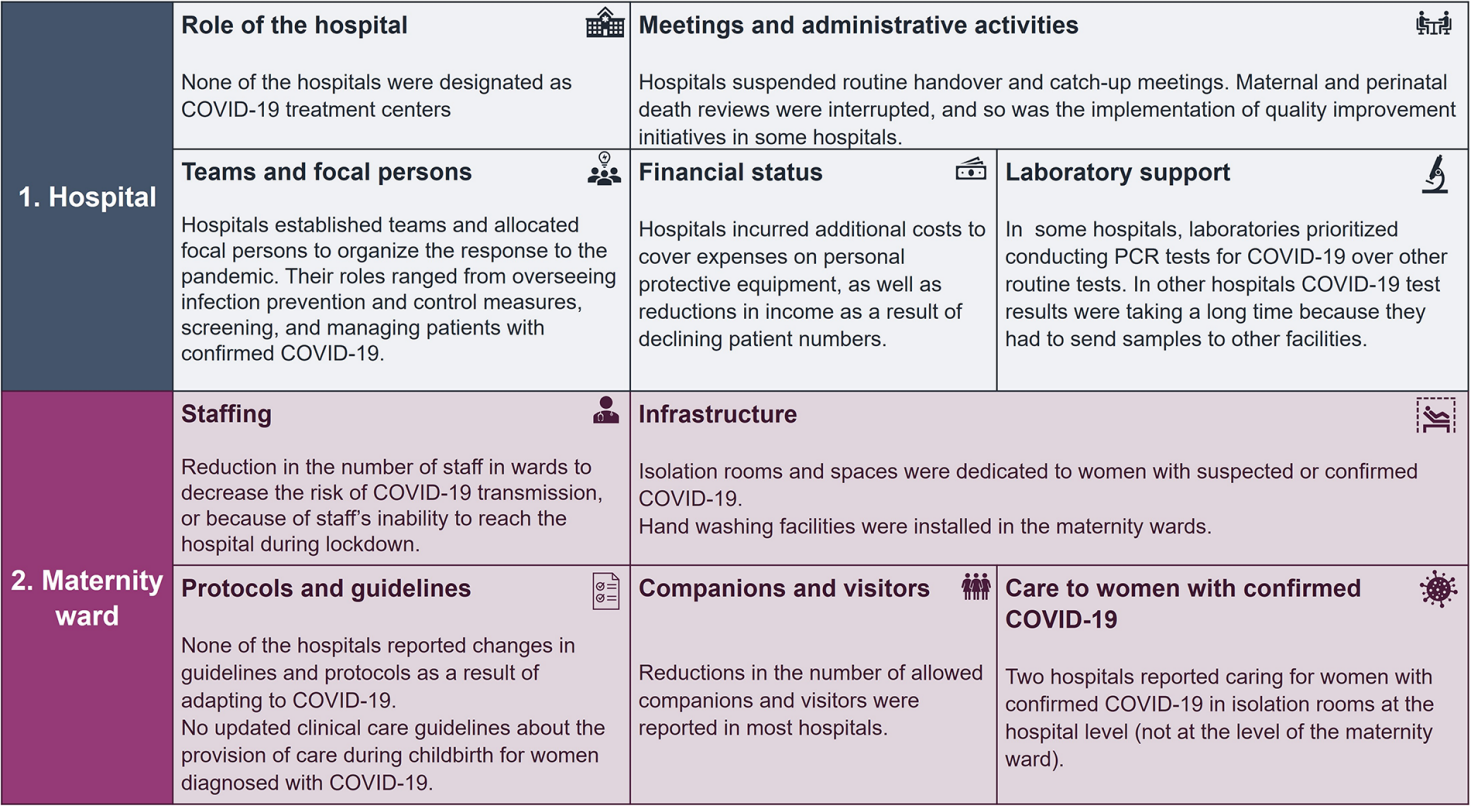
Summary of the categories of management and organization of health services in the 16 hospitals and maternity wards during the COVID-19 pandemic.

Figure 6 summarizes findings of the analysis on management and organization of health service provision during the COVID-19 pandemic, separately at the hospital and maternity ward levels. We explain each category and provide specific examples in the following section. Strengths and lessons-learned are summarized in Box 1.

Box 1 **Strengths and lessons-learned from the country and hospital-level response to the COVID-19 pandemic.**

Our study revealed several points of strength that enabled maternities within hospitals to maintain the provision of care to women and newborns during the pandemic. These were documented at the country/health authority-levels and the hospital/maternity ward levels: National/sub-national level:

- Clear chain of communication and training provided to hospitals regarding screening for and management of COVID-19.
- Health authorities not assigning the referral maternity hospitals as COVID-19 treatment centres could have a been a factor in preventing / reducing fear in the community from seeking care in these facilities which are critical providers of life-saving care for obstetric complications (maintaining trust).

Hospital and maternity ward-level:

- Tapping into existing capacities and skills of staff in the hospital to organize COVID-19 response teams and assign focal-persons.
- No interruptions to maternal and perinatal death review meetings throughout the pandemic.
- Maintaining laboratory capacity to conduct tests related to maternal care provision despite the added new load of conducting COVID-19 tests.
- Ability to retain staff numbers in maternities during lockdowns and throughout the pandemic, with active efforts to protect staff from exposure to infection and from being re-assigned to COVID-19 response.
- Introducing a reward system to compensate maternity staff who were screening for and/or managing patients with COVID-19.
- Dedicating isolation rooms specific to pregnant/birthing/postpartum women with suspected or confirmed COVID-19 despite space shortages in some facilities and wards.
- Introducing/expanding hand washing and disinfection facilities in the maternity ward, placing these more conveniently near points of care, ensuring continuity of running water, soap and hand disinfectant supply.

#### 3.2.1. Hospital-level

##### 3.2.1.1. The “role” of the hospital in the response to the COVID-19 pandemic

None of the hospitals included in this study were assigned as COVID-19 treatment centers. In one hospital in Benin, the hospital management reported negotiating with health authorities regarding the decision to designate the hospital as a COVID-19 referral center.

##### 3.2.1.2. Teams and focal persons managing COVID-19 at the hospital level

Hospitals that established COVID-19 response teams and focal persons operationalized them differently. COVID-19 management teams in Benin and Tanzania had various tasks, including the implementation of infection prevention and control (IPC) measures, i.e., ensuring use of hand-washing devices at the hospital entrance, and raising awareness among patients and healthcare providers to encourage adherence to IPC. Other responsibilities were to ensure screening patients for COVID-19 symptoms, including temperature screening and polymerase chain reaction (PCR) tests, and to manage COVID-19 positive cases at the hospital, or to care for them temporarily before their transfer to designated treatment hospitals. In one Tanzanian hospital, the team was also in charge of handling the bodies of COVID-19 deaths. In one hospital in Malawi the environmental health officer was assigned as a COVID-19 focal person. The district health office (DHO) communicated with the hospitals through meetings regarding the management of COVID-19 patients and provided training to healthcare providers on managing them. None of the hospitals in Uganda reported allocating teams/focal persons responding to COVID-19.

##### 3.2.1.3. Meetings and hospital administrative activities

Some hospitals reported disruptions to administrative and supervisory activities during the COVID-19 pandemic. In one hospital in Malawi, supervisory meetings from the DHO were suspended starting in April 2020. Morning handover meetings and daily catch-ups were suspended in two hospitals in Malawi, and one in Uganda. While staff meetings were suspended, general board meetings continued without changes in MW2.

Similarly, meetings for holding maternal or perinatal death reviews either decreased in frequency or were interrupted altogether during the pandemic in many hospitals, with the exception of Tanzania. In one hospital in Malawi, maternal death audits continued, but were no longer attended by representatives of the DHO.

One hospital in Benin reported that QI initiatives were interrupted during the six months preceding data collection. This hospital had two ongoing initiatives since 2016; on awareness raising among women, and on specific trainings for providers.

##### 3.2.1.4. Hospital financial status

Financial and budgetary changes during the COVID-19 pandemic varied between hospitals and countries. Two hospitals, one in Benin and one in Uganda had increased costs and budget overruns as a result of purchasing personal protective equipment (PPE) and disinfectant. The same hospital in Uganda reported a decline in income resulting from the reduction in patient numbers. Two hospitals in Tanzania reported delays and irregular schedules of funding transfer from the basket fund, although it was not clear whether this was linked to the pandemic. No budgetary changes were reported in Malawi.

##### 3.2.1.5. Laboratory support

During the COVID-19 pandemic, some adaptations in laboratory capacity and support in hospitals were reported, although not uniformly in all settings. In Benin, none of the four hospitals reported changes in laboratory capacity and ability to conduct routine tests related to maternal care provision. Two hospitals in Tanzania reported receiving viral transport media for collecting and transporting samples from COVID-19 suspected patients. Another hospital in Tanzania reported a decrease in patients needing laboratory services during the study period due to a decrease in overall utilization in this hospital. In Malawi, hospitals noted a decline in support from laboratory staff as some were diagnosed with COVID-19 and went into quarantine, and others were covering shifts to conduct COVID-19 screening and collect samples on the border. In two hospitals in Malawi, PCR tests were sent to another facility for analysis, adding delays in receiving results and managing patients, and one hospital resorted to rapid tests. One hospital noted that laboratory equipment was prioritized for COVID-19 tests leading to delays in other tests (e.g., tuberculosis). No changes in the availability of support from laboratories were reported in Benin and Uganda.

#### 3.2.2. Maternity ward-level

##### 3.2.2.1. Staffing

Implications of the COVID-19 pandemic on staff availability in the maternity ward varied between hospitals and countries. None of the hospitals in Benin and Tanzania reported changes in maternity ward staffing levels due to the pandemic. In Malawi, three hospitals noted that maternity staff were divided in groups and were working during alternate weeks. Reductions in staff numbers were only noted in Uganda, either because of health workers' inability to travel during lockdown or because staff numbers per shift were reduced with the introduction of new schedules. In terms of compensation, only one hospital in Malawi noted that staff received payment when screening/treating COVID-19 suspected/confirmed cases.

##### 3.2.2.2. Infrastructure

In terms of infrastructure and care organization, the most commonly reported adaptation was dedicating an isolation ward for women with suspected COVID-19 in the maternity before their referral to treatment centers. One hospital in Uganda and one in Malawi reported designating isolation rooms within the maternity ward, specifically a delivery room for isolating pregnant women with suspected COVID-19. Additionally, five hospitals reported having an isolation room for the hospital in general (not specific to the maternity ward). In Malawi, a hospital dedicated an ambulance for transporting patients suspected with COVID-19. There was a specific triage area dedicated to screening for COVID-19 among patients in one of the hospitals in Benin. Another commonly mentioned change was installing hand washing facilities. In one hospital in Uganda, a hand washing facility was placed at the entrance of the laboratory and hand sanitizers were permanently made available.

##### 3.2.2.3. Protocols and components of maternity care

###### 3.2.2.3.1. Protocols and guidelines

None of the participating hospitals reported formal changes in written clinical care guidelines or protocols as a result of adapting to COVID-19. Additionally, none of the hospitals reported locally updated clinical care guidelines for providing childbirth care, neither received those from authorities nor from other external organizations. All hospitals in Malawi and Uganda, and two hospitals in Benin reported that maternity ward providers received training on COVID-19 during the six months preceding data collection.

###### 3.2.2.3.2. Companionship and visitor policy

Changes in policies on companions and visitors were commonly reported by hospitals. Ten of the 16 hospitals (4 in Benin, one in Tanzania, one in Malawi and 4 in Uganda) noted that the number of allowed persons accompanying women during admission, birth, or visitors during the postpartum period, was reduced during the COVID-19 pandemic. In five of these hospitals (4 in Benin and 1 in Uganda), companions and visitors were required to abide by IPC measures, wear a facemask and wash hands. One hospital in Malawi which previously allowed one birth companion per woman, completely banned companions during the study period, and another hospital only allowed women with complications to have female companions. Other hospitals did not allow companions during labor and birth before the pandemic, and did not report changes to their policy during the study period. In three hospitals in Tanzania where labor companions were not allowed, persons accompanying women to the hospital were instructed to wait outside the hospital, far away from the maternity ward. Data on birth companions and visitors was missing from one hospital in Malawi.

###### 3.2.2.3.3. Care to women with suspected or confirmed COVID-19

Two of the 16 hospitals reported having treated pregnant women with confirmed COVID-19 (two cases in one hospital in Tanzania and six cases in one hospital in Uganda) by the time of data collection. In the Tanzania hospital, women with suspected COVID-19 were isolated and received treatment in a room or ward, designated at the level of the hospital, including after they receive positive PCR test results. In the hospital in Uganda, women with suspected COVID-19 were admitted in a “side room” where samples for the PCR test were taken. If the results, which took three days to come out, were positive, the woman was transferred to an on-site isolation ward. In all the remaining hospitals, women with suspected COVID-19 were managed in an isolation area or room, where the PCR test samples are taken. Women with a positive test result were transferred to COVID-19 treatment centers/hospitals. Two hospitals in Malawi reported that in case of receiving women with suspected/confirmed COVID-19, the women would be isolated together with the newborn (no separation) and that breastfeeding would be encouraged. Nonetheless, these protocols were not applied in these hospitals since they did not report providing care to any pregnant/laboring women with confirmed COVID-19 during the study period.

## 4. Discussion

This paper documents the response to the COVID-19 pandemic in 16 referral hospitals and maternity wards in four countries of sub-Saharan African countries. The results showed variations in the approach adopted in the four countries, as well as variations between hospitals within the same country. Adaptations implemented included allocating focal teams at the hospital level for the COVID-19 response, shifting staffing schedules, designating COVID-19 triage zones and isolation areas and reducing the allowed number of visitors and companions of women giving birth in those hospitals. Interruptions to usual functioning of the hospitals included delayed or cancelled supervisory activities and/or maternal death review meetings and quality improvement initiatives. Budgetary implications involved increased spending and decreased revenues. None of the 16 hospitals were closed or (partly) converted to COVID-19 treatment centers until the study period.

Routine data trends were also extremely context-specific and trends varied by countries and hospitals. The number of ANC visits and facility-based childbirths were not affected to a large extent in Benin hospitals, whereas in Tanzania and Malawi we observed declines that started before the onset of the pandemic in most hospitals. On the other hand, Shapira et al. in their interrupted time-series analysis of national-level data, show a significant decline in ANC and facility deliveries in Malawi, while assuming an interruption date in March 2020 (7). Our analysis shows that in some cases the timing of onset of declines in service use preceded the onset of the pandemic. This could be a result of early fear of COVID-19 in the community due to influence of the international and local media coverage about the pandemic. Another explanation could be that various factors interacting at the health system level beyond the COVID-19 pandemic influence patterns in use of maternal healthcare services. In some Ugandan hospitals, declines in utilization were noted and coincided with the onset of the “movement restriction” requirement. Previous studies in Uganda also showed declines in ANC attendance and facility-based childbirths during the lockdown (12, 35). Periodic increases in rates of maternal deaths were noted in Benin and Uganda (in Benin coinciding with and following restriction measures and in Uganda coinciding with the second wave of the pandemic and the application of movement restrictions). Higher rates of stillbirths were observed in Benin and Uganda in the last two quarters of 2020 compared to 2019.

Our comparative analysis shows that the way in which utilization of maternal care fluctuated during the COVID-19 pandemic appeared to be more closely related to national restriction measures than the COVID-19 epidemiological situation. In Malawi, neither strict movement restrictions nor public transportation bans were introduced, and facility-based childbirths remained stable despite a peak in confirmed COVID-19 cases between June and September 2020. On the other hand, in Uganda there was a small number of confirmed COVID-19 cases in May-June 2020, but the timing of the sudden and strict lockdown was reflected in declines in attendance to both outpatient ANC and inpatient childbirth care. Although the study hospitals reported continuing service provision and not closing, the lockdown seems to negatively influence accessibility to care. This decline in service use is alarming and might be linked to deterioration in maternal and perinatal health outcomes. This was observed in Uganda whereby an increase in maternal mortality was noted in the fourth quarter of 2020 in our study. Additionally, the Ebola outbreak in Sierra Leone was associated with a similar decline in service utilization of ANC and institutional births were accompanied with increases in maternal mortality and stillbirths (36). Policies that reduce accessibility and availability of essential care should be avoided in order to prevent the deterioration in maternal and perinatal health outcomes.

The COVID-19 pandemic is associated with higher rates of stillbirths and maternal deaths compared to before the pandemic (10, 37). Public health decision-makers at the national level should have included pregnant women's healthcare needs as a priority during the planning and response to the COVID-19 pandemic. Such considerations could have avoided unnecessary interruptions in the continuum of maternal and newborn care and prevented the deterioration of perinatal health outcomes, particularly in countries that bear the greater part of this burden. Additionally, health systems' ability to capture changes in maternal and newborn health outcomes is compromised by undocumented deaths that occur outside health facilities (38), issues with data quality (some were documented through this project), and interruptions in data collection and monitoring during the COVID-19 pandemic. Efforts to strengthen the collection of routine data and ensure their quality through regular and stable monitoring systems should be prioritized. This will allow to leverage the value of these data in order to foster preparedness for future health system shocks.

Lockdowns not only affected care utilization, but also care provision, partly through the availability of healthcare providers. Uganda, the country with the strictest measures in our study, is the only country in our study in which maternity wards reported shortages of healthcare providers. Curfews and lockdowns made it difficult for healthcare workers in Uganda, and in other LMICs, to reach their workplace, therefore leading to changes in shift schedules, longer working hours, and burnout (2, 39). This effect exacerbates already severe pre-existing shortages in the health workforce and has negative implications on the quality of maternity care and maternal health outcomes (40).

On the other hand, the majority of maternity healthcare providers at the participating hospitals received training on COVID-19, and in Malawi, there were active efforts to protect the workforce from infection with SARS-CoV-2 through implementing new schedules that reduce contact between staff. Maternity healthcare providers must be prioritized in the response and planning to the COVID-19 pandemic and initiatives such as offering them compensations for managing patients with COVID-19 (reported in a hospital in Malawi) should be encouraged (41).

Another challenge faced by maternity healthcare providers during the COVID-19 pandemic is the lack of knowledge and formal clinical care guidelines for the management of women confirmed with COVID-19 (2, 11). In our study, none of the hospitals reported adopting or updating clinical guidelines for the management of pregnant women with suspected or confirmed COVID-19, although two hospitals reported managing such patients without any official changes to the pre-existing guidelines and protocols. This can be an indication of potentially protecting practices that ensured quality of care, such as encouraging breastfeeding and non-separation of newborn, which were recommended by WHO (23). One of the reported adaptations to care provision was reducing the allowed number of companions and visitors in hospitals. This could indicate that some women did not have access to a companion of choice during the intrapartum period, which contradicts with WHO recommendations (42).

However, most of the study hospitals did not allow birth companions for reasons of privacy and lack of space before the COVID-19 pandemic, so effectively there appeared to have been little change during the pandemic to women's labor and birth companionship. The few hospitals that allow birth companions, enforced infection prevention measures on companions and visitors to reduce the risk of the spread of COVID-19. Additionally, reduction of visitors might mean postpartum women did not have access to support provided by visitors, including meals, laundry, personal hygiene such as bathing self and the baby, and emotional support. The COVID-19 pandemic, and the accompanying mitigation measures have threatened the provision of respectful maternity care in many settings (4). Advocacy should be strengthened to ensure that women and newborns be treated respectfully during and beyond the COVID-19 pandemic.

Aside from interrupting care utilization and provision, the COVID-19 pandemic also disrupted regular monitoring and quality improvement activities. Maternal death reviews were stopped or delayed in all countries included in our study with the exception of Tanzania. This can be a result of multiple factors, including the relatively denialist response to the pandemic in Tanzania during the study period (43). As previously mentioned, such interruption compromises the capacity of hospitals to leverage the value of data systems in monitoring and evaluation and to generate evidence-based policies for emergency preparedness. Emergency preparedness and response planning should be incorporated in all quality improvement initiatives, including maternal death reviews. Continuing maternal death surveillance and response systems during health system shocks, including the COVID-19 pandemic, is of critical importance. Surveillance ensures adequate identification of causes of maternal deaths, including identifying the impact of newly emerging infectious diseases on maternal mortality, and issuing recommendations to avert any negative consequences and improve quality of care.

Many of the applied adaptations and changes were related to infrastructure and resource availability, and varied between hospitals in the same country. For example, the financial impact of the pandemic fell on different entities depending if the hospital is operated by a public or private authority. In Uganda, additional costs resulting from the purchasing of personal protective equipment and conducting PCR tests had to be met by the hospital management in private facilities, and were thus transferred to patient bills. This in turn contributed to driving patients away from the private sector, subsequently reducing the generated income and creating a vicious cycle of budgetary deficit (12). Another example is the lack of laboratory infrastructure to support the rapid issuing of PCR test results for detecting the SARS-CoV-2 virus among women who are pregnant or in labour. This led many maternity wards in our study to send samples for analysis to other institutions, which delayed the test results even further—reaching up to three days in some hospitals. Rapid testing capacity should be strengthened, particularly in maternity wards, as any small delay in care provision can have devastating effects on perinatal health outcomes.

This study's strengths lie in the comparative approach adopted in the synthesis of the findings between and within the four countries. The integration of the results from two data sources allowed for a coherent interpretation of the findings and supporting the evidence with qualitative and quantitative data. Gathering data on adaptations made during the COVID-19 pandemic at the level of the hospital and the maternity ward is another strength of this study as it provides information on similarities and/or differences between the two levels. Additionally, this study builds on a baseline assessment of a large intervention, which showed a flexibility in planning despite difficulties of data collection during the pandemic.

The limitations of this study include combining routine data indicators from four hospitals per country which could have masked hospital-level trends (Supplementary Fle S3). Referral hospitals represent complex adaptive systems, and reactions to health system shocks, such as the COVID-19 pandemic, take shape differently between different hospitals (17, 44, 45). Nonetheless, the aggregation helped in the interpretation of the findings when fluctuations at the hospital-level were not clear, particularly with rare outcomes such as maternal mortality. Additionally, this aggregation allows a bigger sample size and represents the case of four large referral hospitals with non-overlapping catchment areas in each country. One of our study's limitations was the missingness of responses to the open-ended questions in the HFA for some hospitals. More complete information was available from the secondary country-level data (e.g., dates and durations of implemented mitigation measures at the country level) and these country-level policies apply to all the hospitals in each country. The aggregation of service statistics at the country-level thus allowed us to triangulate between the country-level data and the quantitative data, which supported our interpretation of the findings. We also acknowledge that collecting the open-ended responses with the HFA could have introduced a level of information bias, as the information is self-reported by respondents working at the selected hospitals. Another limitation is that routine data were extracted from HMIS and therefore quality assurance over these data were difficult to conduct. A unified perinatal data collection system in hospitals could improve data quality and inform quality improvement initiatives; the development and implementation of such a system is part of the ALERT project, and will be used to collect the data for intervention evaluation (25). Last, it is difficult to restrict the explanation of certain routine data trends to the COVID-19 pandemic and its accompanying mitigation measures alone. Some trends in routine data, such as the decline in ANC consultations and the increase in stillbirths, were ongoing even before the onset of the pandemic. Complex factors contribute to such increases and are difficult to identify within the scope of this study and should be explored further.

## 5. Conclusion

Our study documents that disruptions during the COVID-19 pandemic go beyond the relatively frequently described issues with access to and utilization of maternity care, to include complex issues related to hospital governance and financing, resource availability, modality of care provision, disruptions of certain aspects of care quality, and unnecessary interruptions to routine quality improvement activities. The documented within and between country differences in the response to the pandemic demonstrates the complexity of the issue at hand, and is an example of the difficulty of establishing a unified response at the national and international level. This highlights the importance of contextualized solutions and adaptations in response to the ongoing COVID-19 pandemic, and future shocks to the health system.

## Data Availability

The datasets presented in this article are not readily available because they cannot be shared for ethical/privacy reasons. The data underlying this article cannot be shared publicly in order to protect the privacy of hospitals and the hospital staff who participated in the study. The data will be shared on reasonable request to the lead data manager of ALERT. Requests to access the datasets should be directed to Kristi Sidney Annerstedt, kristi.sidney@ki.se.
