# Supplementary material for: Provision and utilization of maternal health services during the COVID-19 pandemic in 16 hospitals in sub-Saharan Africa
Source: Front Glob Womens Health. 2023 Oct 31;4:1192473. doi: 10.3389/fgwh.2023.1192473 (PMC10644718; doi:10.3389/fgwh.2023.1192473)
Supplement: Supplementary file 1 [file Table1.pdf]

Supplementary table 1. Characteristics of the 16 participating hospitals and maternity wards before the COVID-19 pandemic

Country	BENIN				MALAWI			
**Hospital**	**BN1**	**BN2**	**BN3**	**BN4**	**MW1**	**MW2**	**MW3**	**MW4**
**Hospital characteristics**								
Operating authority	Public	Public	Public	Private	Public	Private	Public	Public
Established between	2000-2005	1985-1990	1960-1965	1995-2000	1985-1990	1955-1960	2000-2005	1985-1990
Total number of beds	134	362	648	68	222	290	100	293
Catchment population (in 000s)	410	2805	11 880	1720	630	250	550	685
**Maternity services provided**								
Routine outpatient ANC	Yes	Yes	Yes	Yes	Yes	Yes	Yes	Yes
Childbirth/caesarean sections	Yes	Yes	Yes	Yes	Yes	Yes	Yes	Yes
Routine outpatient PNC	Yes	Yes	Yes	Yes	Yes	Yes	Yes	Yes
Private maternity care[†]	No	No	No	No	No	Yes	No	No
Dedicated theatre for caesarean section	Yes	Yes	Yes	Yes	No	No	Yes	No
HDU or ICU for women	Yes	Yes	Yes	No	Yes	Yes	No	No
NICU (year NICU added)	Yes (2019)	Yes (1990)	Yes (1978)	Yes (1996)	No	No	No	No
**Maternity ward characteristics**								
Number of outpatient ANC visits (2019)	2588	5446	4615	7045	5885	4625	12 336	7791
Number of deliveries (2019)	2088	5740	2605	2186	7363	3276	6373	7791
Perinatal mortality rate* (2019)	--	78	--	48	51	17	34	19
Number of beds for second stage of labour	3	10	4	4	4	6	5	9
Frequency of women exceeding labour beds	Once a week	Every day	Once a week	Every day	Once a month	Few times per year	--	--
Number of medical professionals working per shift on weekday daytime**	6	7	18	3	14	10	13	--
LOS after uncomplicated vaginal birth as per usual practice	--	1 day	24 hours	24 hours	--	24 hours	1 day	24 hours
LOS (minimum) after caesarean section as per usual practice	7 days	3 days	4 days	3 days	3 days	3 days	3 days	3 days
User fees for maternity care	Yes (with exception of caesarean sections)	Yes (with exception of caesarean sections)	Yes (with exception of caesarean sections)	Yes (with exception of caesarean sections)	No	Yes	No	No

Supplementary table 1 (cont.) Characteristics of the 16 participating hospitals and maternity wards before the COVID-19 pandemic

**Country**	**TANZANIA**				**UGANDA**			
**Hospital**	**TZ1**	**TZ2**	**TZ3**	**TZ4**	**UG1**	**UG2**	**UG3**	**UG4**
**Hospital characteristics**								
Operating authority	Private	Public	Public	Public	Public	Private	Public	Public
Established between	1925-1930	1950-1955	1950-1955	1955-1960	1930-1935	1910-1915	1965-1970	1965-1970
Total number of beds	306	207	138	280	500	160	100	100
Catchment population (in 000s)	160	28	162	95	4000	20	2000	400
**Maternity services and infrastructure**								
Routine outpatient ANC	Yes	Yes	Yes	Yes	Yes	Yes	Yes	Yes
Childbirth/caesarean sections	Yes	Yes	Yes	Yes	Yes	Yes	Yes	Yes
Routine outpatient PNC	Yes	Yes	Yes	Yes	Yes	Yes	Yes	Yes
Private maternity care	No	No	No	No	Yes	Yes	No	No
Dedicated theatre for caesarean section	No	No	No	No	No	No	No	No
HDU or ICU for women	No	No	No	No	No	No	No	No
NICU (year NICU added)	Yes (2013)	No	Yes (2012)	Yes (2015)	Yes (2020)	Yes^ (2016)	Yes (2020)	Yes^ (2016)
**Maternity ward characteristics**								
Number of outpatient ANC visits (2019)	4469	5108	3770	--	10 951	5855	15 622	10 070
Number of deliveries (2019)	2048	4788	2242	2981	6070	1265	7169	3531
Perinatal mortality rate* (2019)	49	40	44	81	62	115	52	54
Number of beds for 2nd stage of labour	4	4	6	5	6	6	6	2
Frequency of women exceeding labour beds	Few times per year	Once a week	Once a week	Few times per year	Few times per year	Few times per year	Every day	Every day
Number of medical professionals working per shift on weekday daytime**	3	3	3	5	6	6	4	2
LOS after uncomplicated vaginal birth as per usual practice	12 hours	12 hours	12 hours	12-24 hours; discharge rounds take place once daily	12 hours	24 hours	12 hours	12 hours
LOS (minimum) after caesarean section as per usual practice	48 hours	48 hours	72 hours	48 hours	72 hours	3 days	72 hours	4 days
User fees for maternity care	Yes	No, but women asked to bring materials/supplies	No, but women asked to bring materials/supplies	No, but women asked to bring materials/supplies	No (with exception of patients in private maternity ward)	Yes	No	No

†Private maternity care refers to the availability of an optional private services such as a private labour ward or private room for postnatal care. The package could include supplementary services such as closer monitoring and provision of drinking water.
*Perinatal mortality rate is calculated as the number of stillbirths and early neonatal deaths per 1,000 births
**Medical professionals include gynaecologists, midwives, nurses, anaesthetists, interns and doctors in training, clinical officers, medical officers, assistant clinical officers, patient attendant.
^Neonatal special care unit
Abbreviations: Antenatal care (ANC); Length of stay (LOS); Postnatal care (PNC); High-dependency unit (HDU); Intensive care unit (ICU); Neonatal intensive care unit (NICU); Human immunodeficiency virus (HIV)
(--) indicates missing data
