# Supplementary material for: Provision and utilization of maternal health services during the COVID-19 pandemic in 16 hospitals in sub-Saharan Africa
Source: Front Glob Womens Health. 2023 Oct 31;4:1192473. doi: 10.3389/fgwh.2023.1192473 (PMC10644718; doi:10.3389/fgwh.2023.1192473)
Supplement: Supplementary file 2 [file Datasheet1.pdf]

Benin

	January	February	March	April	May	June	July	August	September	October	November	December
International travel control												
Movement restrictions												
Stay at home requirement												
Public transport restriction*												
Workplace closure												
School closure												

*Reduced number of allowed passengers on public transport

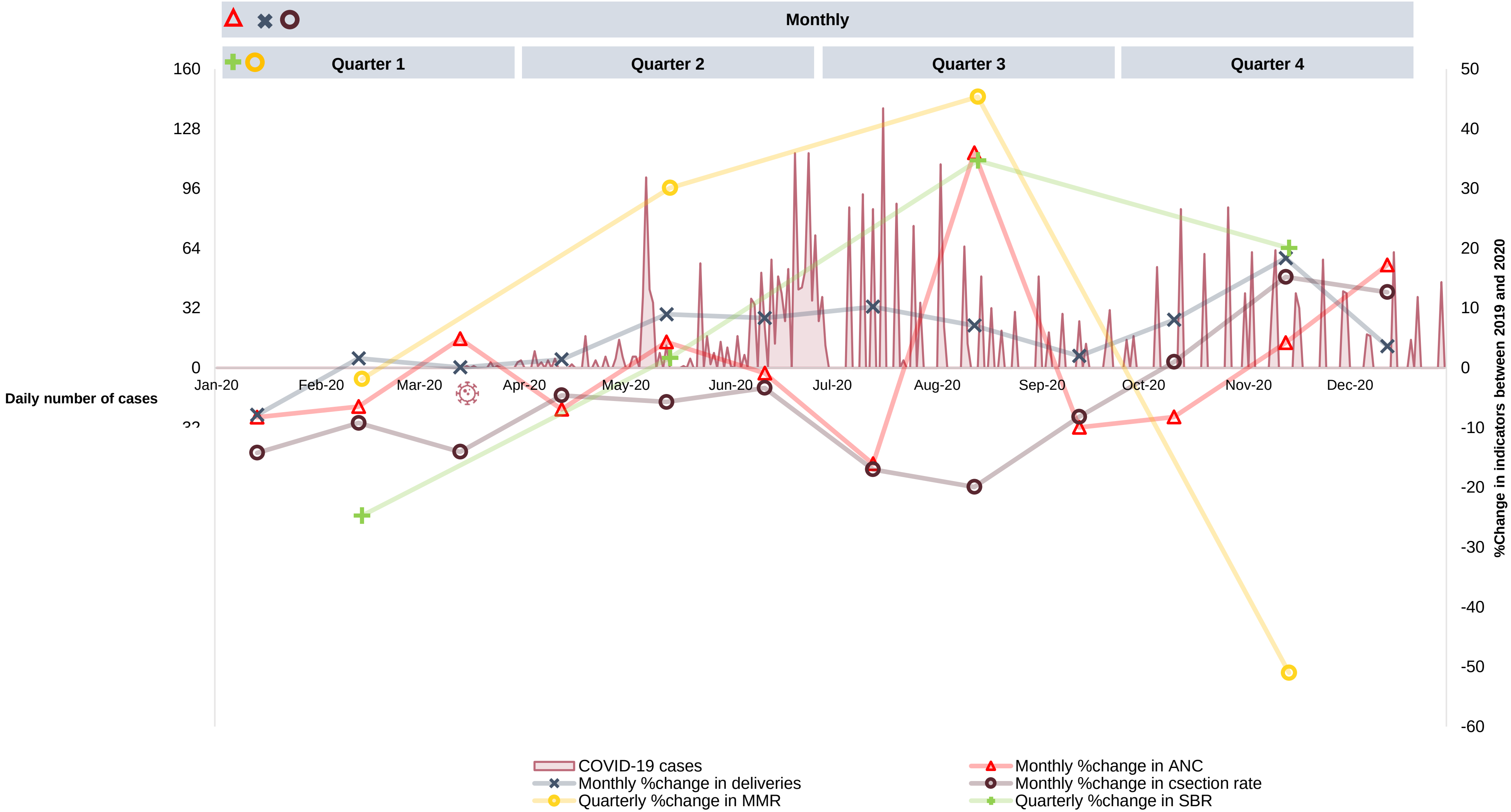

Required measure / Total border closure — Recommended measure / Screening or quarantining required on arrival — First COVID-19 case

Malawi

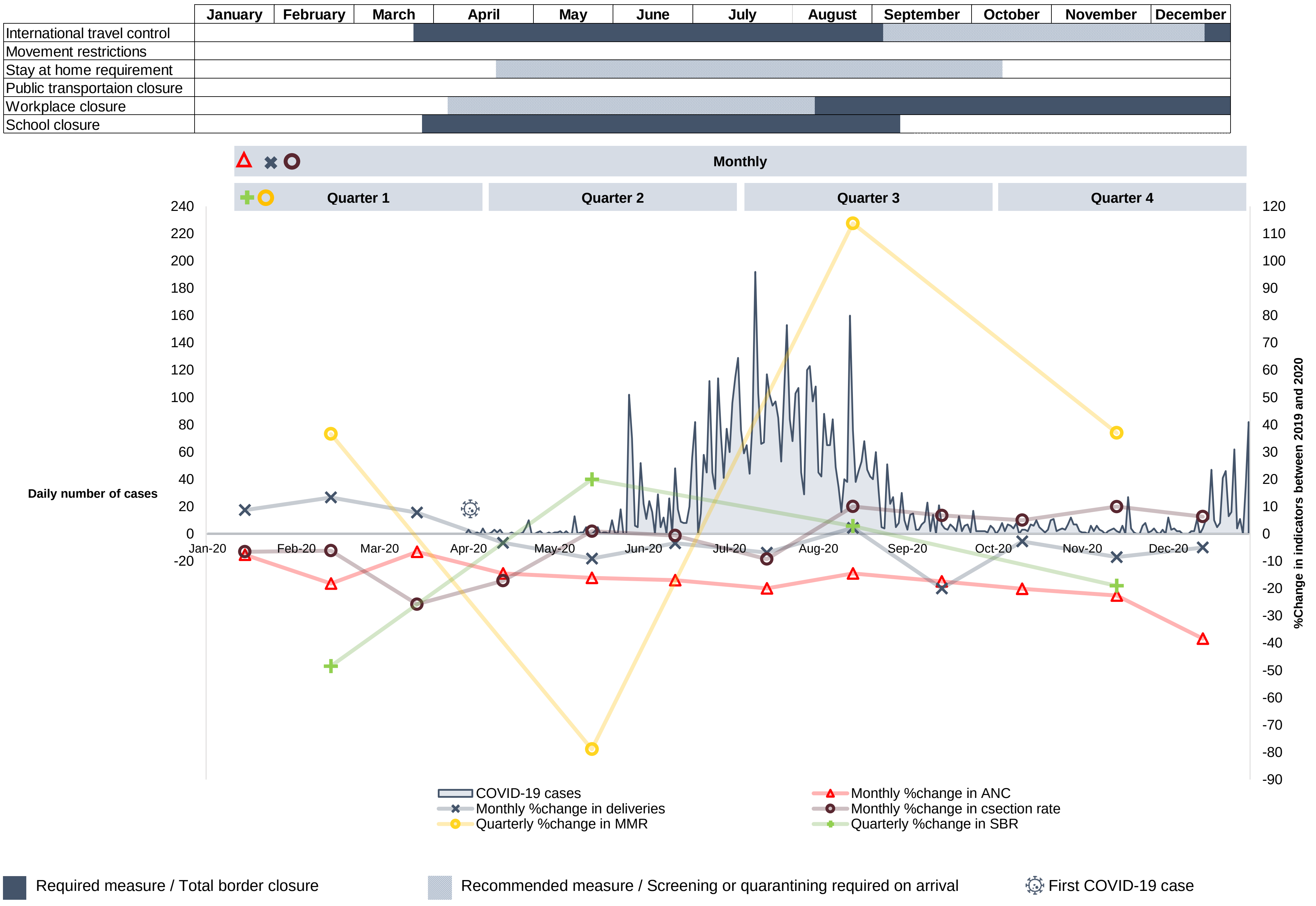

January
February
March
April
May
June
July
August
September
October
November
December
International travel control
Movement restrictions
Stay at home requirement
Public transportaion closure
Workplace closure
School closure
Monthly
Quarter 1
Quarter 2
Quarter 3
Quarter 4
Daily number of cases
240
220
200
180
160
140
120
100
80
60
40
20
0
-20
Jan-20
Feb-20
Mar-20
Apr-20
May-20
Jun-20
Jul-20
Aug-20
Sep-20
Oct-20
Nov-20
Dec-20
120
110
100
90
80
70
60
50
40
30
20
10
0
-10
-20
-30
-40
-50
-60
-70
-80
-90
%Change in indicators between 2019 and 2020
COVID-19 cases
Monthly %change in deliveries
Quarterly %change in MMR
Monthly %change in ANC
Monthly %change in csection rate
Quarterly %change in SBR
Required measure / Total border closure
Recommended measure / Screening or quarantining required on arrival
First COVID-19 case

Tanzania

	January	February	March	April	May*	June	July	August	September	October	November	December
International travel control												
Movement restrictions												
Stay at home requirement												
Public transportaion closure												
Workplace closure												
School closure												

*Number of COVID-19 cases not available/reported beyond this date

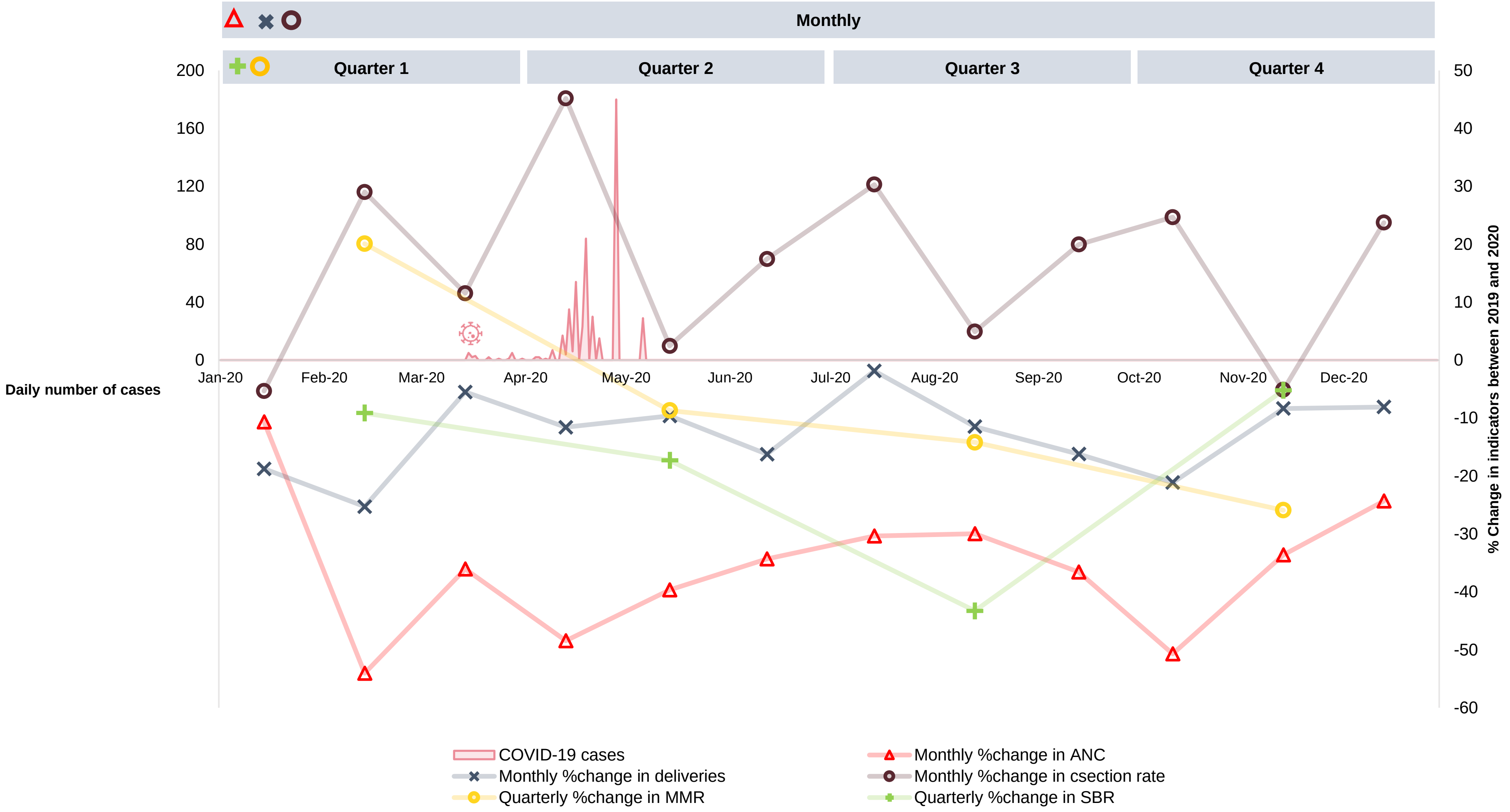

Required measure / Total border closure

Recommended measure / Screening or quarantining required on arrival

First COVID-19 case

Uganda

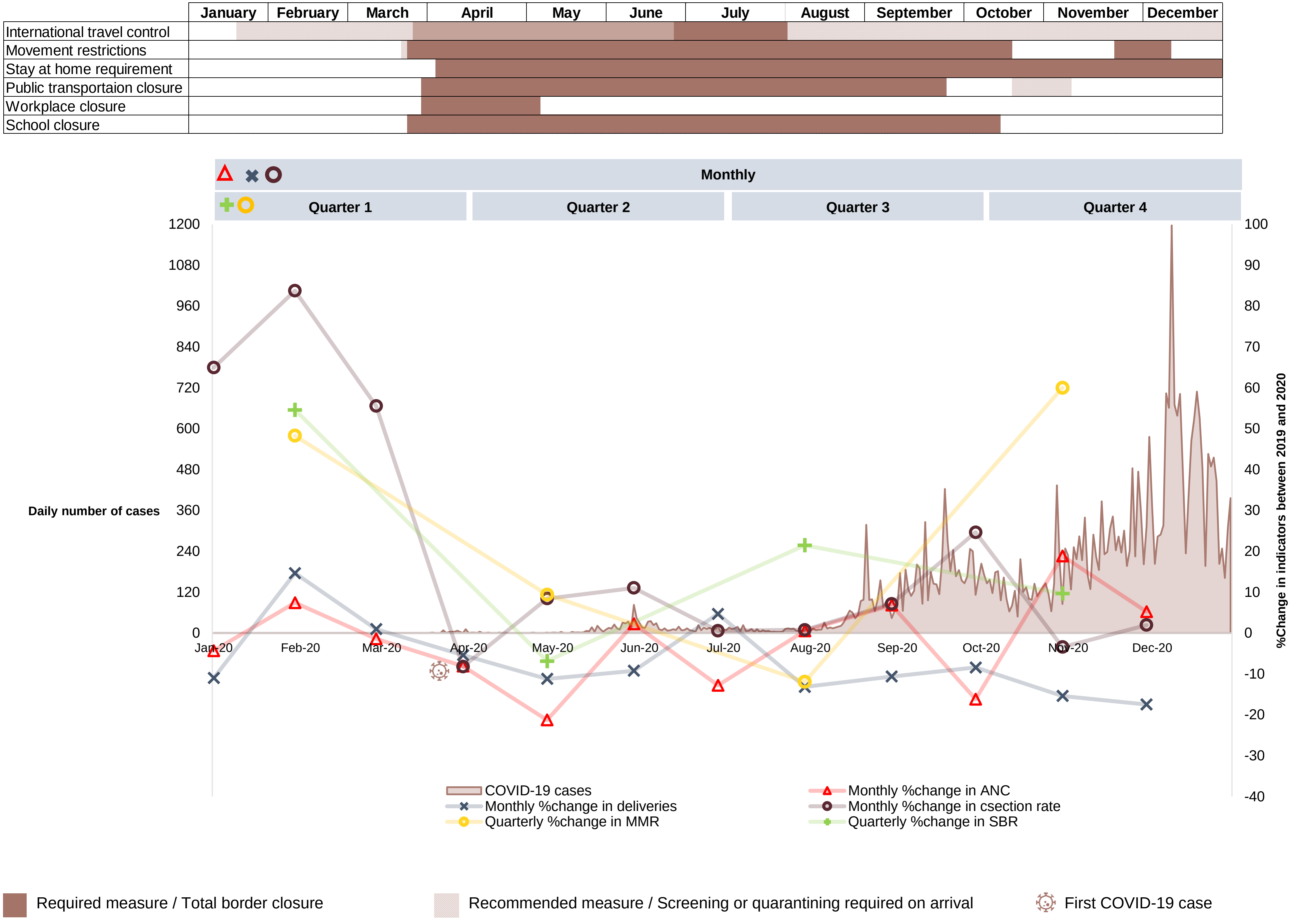

January
February
March
April
May
June
July
August
September
October
November
December
International travel control
Movement restrictions
Stay at home requirement
Public transportaion closure
Workplace closure
School closure
Monthly
Quarter 1
Quarter 2
Quarter 3
Quarter 4
Daily number of cases
1200
1080
960
840
720
600
480
360
240
120
0
100
90
80
70
60
50
40
30
20
10
0
-10
-20
-30
-40
%Change in indicators between 2019 and 2020
Jan-20
Feb-20
Mar-20
Apr-20
May-20
Jun-20
Jul-20
Aug-20
Sep-20
Oct-20
Nov-20
Dec-20
COVID-19 cases
Monthly %change in deliveries
Quarterly %change in MMR
Monthly %change in ANC
Monthly %change in csection rate
Quarterly %change in SBR
Required measure / Total border closure
Recommended measure / Screening or quarantining required on arrival
First COVID-19 case
