# Supplementary material for: Provision and utilization of maternal health services during the COVID-19 pandemic in 16 hospitals in sub-Saharan Africa
Source: Front Glob Womens Health. 2023 Oct 31;4:1192473. doi: 10.3389/fgwh.2023.1192473 (PMC10644718; doi:10.3389/fgwh.2023.1192473)
Supplement: Supplementary file 3 [file Datasheet2.docx]

| **BN1** |  |
| --- | --- |
| **BN2** |  |
| **BN3** |  |
| **BN4** |  |

**Figure S1.1 - Monthly number of antenatal care visits in the four hospitals in Benin in 2019 and 2020**

| **MW1** |  |
| --- | --- |
| **MW2** |  |
| **MW3** |  |
| **MW4** |  |

**Figure S1.2 - Monthly number of antenatal care visits in the four hospitals in Malawi in 2019 and 2020**

| **TZ1** |  |
| --- | --- |
| **TZ2** |  |
| **TZ3** |  |
| **TZ4** | **NO DATA** |

**Figure S1.3 - Monthly number of antenatal care visits in the four hospitals in Tanzania in 2019 and 2020**

**Figure S1.4 - Monthly number of antenatal care visits in the four hospitals in Uganda in 2019 and 2020**

**Antenatal care visits - Tanzania**

| **UG1** | **NO DATA** |
| --- | --- |
| **UG2** |  |
| **UG3** |  |
| **UG4** |  |

**Antenatal care visits - Uganda**

**Figure S2.1 - Monthly number of deliveries in the four hospitals in Benin in 2019 and 2020**

| **BN1** |  |
| --- | --- |
| **BN2** |  |
| **BN3** |  |
| **BN4** |  |

**Figure S2.2 - Monthly number of deliveries in the four hospitals in Malawi in 2019 and 2020**

| **MW1** |  |
| --- | --- |
| **MW2** |  |
| **MW3** |  |
| **MW4** |  |

**Figure S2.3 - Monthly number of deliveries in the four hospitals in Tanzania in 2019 and 2020**

| **TZ1** |  |
| --- | --- |
| **TZ2** |  |
| **TZ3** |  |
| **TZ4** |  |

**Figure S2.4 - Monthly number of deliveries in the four hospitals in Uganda in 2019 and 2020**

| **UG1** |  |
| --- | --- |
| **UG2** |  |
| **UG3** |  |
| **UG4** |  |

**Figure S3.1 - Monthly numbers and percentages of caesarean section in the four hospitals in Benin in 2019 and 2020**

| **BN1** |  |
| --- | --- |
| **BN2** |  |
| **BN3** |  |
| **BN4** |  |

**Figure S3.2 - Monthly numbers and percentages of caesarean section in the four hospitals in Malawi in 2019 and 2020**

| **MW1** |  |
| --- | --- |
| **MW2** |  |
| **MW3** |  |
| **MW4** |  |

**Figure S3.3 - Monthly numbers and percentages of caesarean section in the four hospitals in Tanzania in 2019 and 2020**

| **TZ1** |  |
| --- | --- |
| **TZ2** |  |
| **TZ3** |  |
| **TZ4** |  |

**Figure S3.4 - Monthly numbers and percentages of caesarean section in the four hospitals in Uganda in 2019 and 2020**

| **UG1** |  |
| --- | --- |
| **UG2** |  |
| **UG3** |  |
| **UG4** |  |

**Figure S5.1 - Quarterly number and rate per 100,000 deliveries of in-facility maternal mortality in the four hospitals in Benin, in 2019 and 2020**

| **BN1** |  |
| --- | --- |
| **BN2** |  |
| **BN3** |  |
| **BN4** |  |

**Figure S5.2 - Quarterly number and rate per 100,000 deliveries of in-facility maternal mortality in the four hospitals in Malawi, in 2019 and 2020**

| **MW1** |  |
| --- | --- |
| **MW2** | No maternal deaths |
| **MW3** |  |
| **MW4** |  |

**Figure S5.3 - Quarterly number and rate per 100,000 deliveries of in-facility maternal mortality in the four hospitals in Tanzania, in 2019 and 2020**

| **TZ1** |  |
| --- | --- |
| **TZ2** |  |
| **TZ3** |  |
| **TZ4** |  |

**Figure S5.4 - Quarterly number and rate per 100,000 deliveries of in-facility maternal mortality in the four hospitals in Uganda, in 2019 and 2020**

| **UG1** |  |
| --- | --- |
| **UG2** |  |
| **UG3** |  |
| **UG4** |  |

**Figure S6.1 - Quarterly number and rate per 1000 deliveries of stillbirths in the four hospitals in Benin, in 2019 and 2020**

| **BN1** |  |
| --- | --- |
| **BN2** |  |
| **BN3** |  |
| **BN4** |  |

**Figure S6.2 - Quarterly number and rate per 1000 deliveries of stillbirths in the four hospitals in Malawi, in 2019 and 2020**

| **MW1** |  |
| --- | --- |
| **MW2** |  |
| **MW3** |  |
| **MW4** |  |

**Figure S6.3 - Quarterly number and rate per 1000 deliveries of stillbirths in the four hospitals in Tanzania, in 2019 and 2020**

| **TZ1** |  |
| --- | --- |
| **TZ2** |  |
| **TZ3** |  |
| **TZ4** |  |

**Figure S6.4 - Quarterly number and rate per 1000 deliveries of stillbirths in the four hospitals in Uganda, in 2019 and 2020**

| **UG1** |  |
| --- | --- |
| **UG2** |  |
| **UG3** |  |
| **UG4** |  |


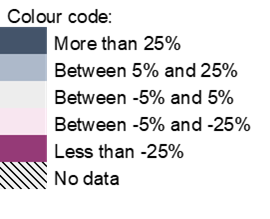
**Figure S7 - Percent change in perinatal health indicators between 2019 and 2020 at the country level and hospital level**

|  | **Antenatal care visits** | **Deliveries** | **Caesarean section (%)** | **Maternal Mortality Rate** | **Stillbirth rate** |
| --- | --- | --- | --- | --- | --- |
| **Benin** | **-0.2** | **5.5** | **-7.0** | **1.8** | **5.2** |
| **BN1** | 4 | 2 | 8 | 11 | -5 |
| **BN2** | **27** | 18 | -17 | -22 | 14 |
| **BN3** | -12 | -6 | 5 | **39** | -1 |
| **BN4** | -15 | -11 | 3 | **57** | -18 |
|  |  |  |  |  |  |
| **Malawi** | **-17.8** | **-2.9** | **-2.4** | **16.7** | **-15.1** |
| **MW1** | **-53** | 1 | -12 | **65** | **-45** |
| **MW2** | -1 | 23 | 3 | 0 | 5 |
| **MW3** | -13 | -10 | -4 | **-78** | 3 |
| **MW4** | -10 | -11 | -5 | **69** | 11 |
|  |  |  |  |  |  |
| **Tanzania** | **-36.8** | **-13.1** | **13.0** | **-7.2** | **-18.1** |
| **TZ1** | **-62** | -3 | **28** | **43** | **-26** |
| **TZ2** | -15 | -13 | 4 | **-56** | -16 |
| **TZ3** | 0 | 0 | 0 | 11 | -2 |
| **TZ4** |  | -21 | 10 | **-50** | -16 |
|  |  |  |  |  |  |
| **Uganda** | **-2.9** | **-6.9** | **12.5** | **24.1** | **19.5** |
| **UG1** |  | -11 | **34** | **91** | **34** |
| **UG2** | -7 | -23 | 16 | **180** | 21 |
| **UG3** | -7 | -11 | 5 | -13 | 24 |
| **UG4** | 13.0 | 15 | 13 | **-53** | -1 |
